# πSPIM: high NA high resolution isotropic light-sheet imaging in cell culture dishes

**DOI:** 10.1038/srep32880

**Published:** 2016-09-13

**Authors:** Patrick Theer, Denitsa Dragneva, Michael Knop

**Affiliations:** 1 Zentrum für Molekulare Biologie der Universität Heidelberg (ZMBH), University of Heidelberg, Im Neuenheimer Feld 282, 69120 Heidelberg, Germany; 2 Deutsches Krebsforschungszentrum (DKFZ), Im Neuenheimer Feld 280, 69120 Heidelberg, Germany

## Abstract

Light-sheet fluorescence microscopy (LSFM), also termed single plane illumination microscopy (SPIM), enables live cell fluorescence imaging with optical sectioning capabilities superior to confocal microscopy and without any out-of-focus exposure of the specimen. However, the need of two objective lenses, one for light-sheet illumination and one for imaging, imposes geometrical constraints that require LSFM setups to be adapted to the specific needs of different types of specimen in order to obtain optimal imaging conditions. Here we demonstrate the use of an oblique light-sheet configuration adapted to provide the highest possible Gaussian beam enabled resolution in LSFM. The oblique light-sheet configuration furthermore enables LSFM imaging at the surface of a cover slip, without the need of specific sample mounting. In addition, the system is compatible with simultaneous high NA wide-field epi-fluorescence imaging of the specimen contained in a glass-bottom cell culture dish. This prevents cumbersome sample mounting and enables rapid screening of large areas of the specimen followed by high-resolution LSFM imaging of selected cells. We demonstrate the application of this microscope for *in toto* imaging of endocytosis in yeast, showing for the first time imaging of all endocytic events of a given cell over a period of >5 minutes with sub-second resolution.

Fluorescence microscopy using light-sheet illumination enables isotropic imaging of living specimen while keeping the illumination-associated damage of the sample low. Light-sheet microscopes are commonly based on an orthogonal arrangement of two objective lenses, one for light-sheet generation and one for emission detection^1^,^2^. While this arrangement derives from the optimum alignment of the light-sheet and detection axes, it imposes several physical constrains entailed by the proximity of the two objective lenses. Most importantly, an orthogonal arrangement limits the choice of objective lenses to pairs whose access angles add up to less than 90° and, more importantly, it restricts the choice of NA opening angles which has a direct impact on sensitivity, resolution, and light-sheet thickness. In addition, it presents major challenges to sample preparation and positioning, as the objective lenses limit access to the area where the light-sheet is positioned. However, an orthogonal arrangement of the objective lenses is not a mandatory requirement for light-sheet microscopy, since it is possible to generate light-sheets at an oblique angle^3^,^4^,^5^, or, with the help of micro-mirrors, even in the focal plane of the light-sheet generating objective^6^,^7^. Oblique and micro-mirror generated light-sheets can ease many of the constraints commonly encountered in light-sheet microscopy but have been almost solely employed in order to dispose one of the objective lenses enabling single objective lens (epi-fluorescence) light-sheet microscopy^3^,^4^,^5^,^8^. Relying on a single lens, however, introduces some new constraints - most prominently on the light-sheet NA which tends to be quite low. In single-lens oblique light-sheet microscopy this is because the available NA has to be split up between light-sheet and detection NA so that there is always a trade-off between light-sheet thickness and detection efficiency^4^. The NA of micro-mirror generated light-sheets is mainly limited by the size of the mirror but complicated by the fact that the 90° folding of the light-sheet plane requires an axial offset between light-sheet and coverslip plane which scales linearly with NA and field-of-view (FOV). Imaging below this offset, i.e., closer to the coverslip leads to beam clipping at the micro mirror^7^ leaving sample features close to the coverslip optically inaccessible. Attempts to circumvent this offset problem by mounting the sample on a raised platform can reduce but not evade this problem and come at the cost of a reduced FOV and elaborate sample mounting. These constraints are often unsatisfactory and in particular for high-resolution single-cell imaging prohibitive. Here we elaborate on the principle of oblique light-sheets in the context of non-orthogonal dual lens arrangements, which are not bound by those single lens problems. We demonstrate an implementation of such a system, termed πSPIM, for high-resolution single-cell imaging. The tested design eliminates all physical constraints between the objective lenses and permits the use of specimen contained on the surface of a glass bottom culture dish. A non-orthogonal arrangement allows for free adjustment of light-sheet angle and NA within the bounds of the free angular range (access angle) of a given detection objective (Fig. 1), with the sum of the opening angles 2(α_NAexc._ + α_NAdet._) ultimately approaching π. πSPIM uses an inverted arrangement of an oil immersion objective lens, which serves for wide-field imaging and for oblique light-sheet generation simultaneously. The detection objective is in an upright position but tilted by an angle close to its access angle (Fig. 1). The angle of the oblique light-sheet as well as its thickness (NA) can be adjusted by varying the lateral offset and width of the illumination beam in the back-focal plane of the illumination objective. With the use of high-NA (≥1.4) oil immersion objective lenses for light-sheet generation, the light-sheet NA can be freely adjusted to ultimately fill all of the angular range left available by the detection objective. Such an arrangement also allows generous access to the specimen and importantly, it permits it to reside on the surface of the cover slip (e.g. adherent cells). This is a prerequisite for simultaneous high-NA wide-field imaging through the illumination objective and thus can be combined with a wide-field set-up for convenient selection of specific areas in the full FOV of the specimen, such as individual cells, for their subsequent analysis using light-sheet imaging. This is particularly useful when light-sheet microscopy is employed to study a rare subpopulation among the cells present in the sample.

## Theory, Results and Discussion

### Resolution in light-sheet microscopy

The resolution in light-sheet fluorescence microscopy (LSFM) is characterized by its effective point-spread function (PSF), which results from the overlap of the light-sheet used for illumination and the PSF of the detection objective lens. The resulting PSF can be calculated as the product of the excitation PSF (*h*_*exc*._), which is proportional to the excitation intensity distribution, and the detection PSF (*h*_det._), which describes the spatial distribution of the detection efficiency:


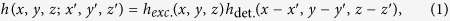


where *x*′, *y*′, *z*′ are the distances from the center of the FOV. For the majority of applications in LSFM the following approximations hold:

the excitation intensity does not vary in the direction perpendicular to the excitation and detection axes, i.e., 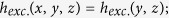


the center of the detection PSF lies in the light-sheet plane i.e. *z*′ = 0;

the variation of the excitation intensity in the light-sheet plane over the lateral extend of *h*_det._, which is on the order of *λ*, is negligible (it is zero along *x*).

Hence, the effective lateral resolution is independent of *h*_*exc*._ (shift invariant) and determined solely by the lateral extend of the detection PSF.

To be generally applicable, we consider here a non-paraxial scalar description for the PSF given (in cylindrical co-ordinates), see refs 9 and 10:


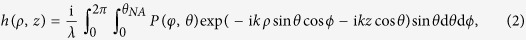


where *y* is the axial co-ordinate, 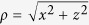 is the radial co-ordinate, *θ* and *φ* are the azimuth and polar angle, respectively, and *P*(*φ*, *θ*) is the apodization function that describes the intensity distribution across the converging wavefront^9^. For objectives fulfilling the sine condition (as microscope objective lenses in general do) 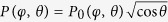, where *P*_0_(*φ*, *θ*) is the pupil function that describes the light intensity distribution in the back-focal plane. For the detection PSF the pupil function depends only on the transmission characteristics of the used objective, which can be considered constant across the pupil 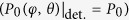 (for well corrected objectives):


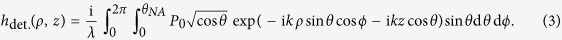


For the excitation PSF the pupil function depends also on the excitation light distribution in the pupil, which is typically illuminated with a focused light-sheet so that the pupil function is zero everywhere except along a single diameter e.g. 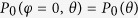 and 0 anywhere else:


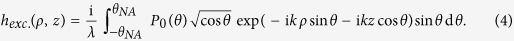


### Oblique light-sheet generation

**Figure 1:**
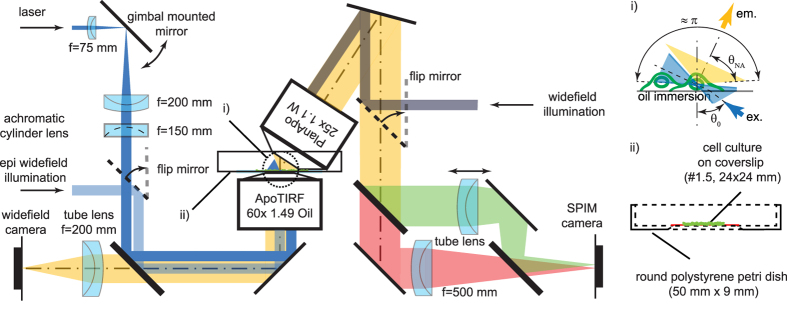
πSPIM set-up. Schematic dual-color πSPIM set-up with an oblique light-sheet produced by off-center passage of the beam through the illumination lens (1.49 ApoTIRF 60×), and the detection lens (1.1 W, 25×) arranged orthogonally to the oblique light-sheet. (i) Close-up of the focal region showing the angular range of the complementing illumination and emission cones. (ii) Mid-plane cross-section of the glass-bottom dish used for sample mounting.

For the generation of an oblique light-sheet (Fig. 1) a non-centrosymmetric pupil function is required, i.e., *P*_0_(*θ*–*θ*_0_). In order for the light-sheet plane to coincides with the detection focal plane at a given tilt angle *θ*_0_, the light-sheet axis in the pupil has to have a lateral offset by *R* = *f sin*(*θ*_0_):


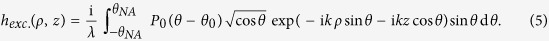


### Minimum focal volume and resolution anisotropy

**Figure 2:**
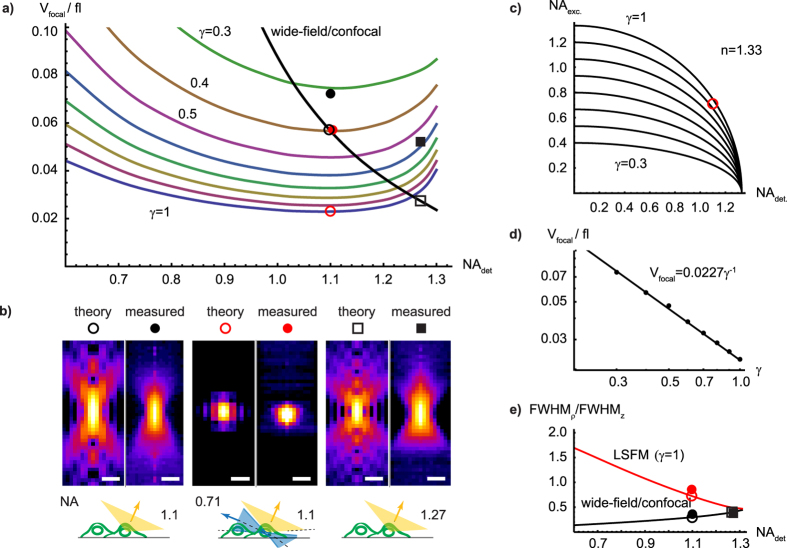
Effective focal volume and anisotropy. (**a**) Effective focal volume element calculated for wide-field/confocal and light-sheet microscopes as a function of detection NA and various angular fill factors *γ*. Symbols indicate theoretical and estimated volumes of the PSFs presented in (b). (**b**) PSF comparison. Theoretical and measured PSF (x-z, log-scale, radially averaged) for the used 1.1 NA detection objective (left panels), the πSPIM with 1.1 detection and 0.71 excitation light-sheet NA (*γ* ≈ 1) (panels in the center). Panels to the right: for purpose of comparison, theoretical and measured PSF of the highest currently available water immersion) NA of 1.27. Bars, 500 nm. Focal volumes are from left to right about 0.057, ***0***.***072***, 0.023, ***0***.***057***, 0.027, ***0***.***052*** fl (theory, ***measured***) and indicated in (a). (**c**) Excitation NA as a function of detection NA and angular fill-factor *γ* for water immersion set-ups (n = 1.33). (**d**) Minimal focal volume as a function of angular fill factor *γ* (**e**) Resolution anisotropy (*FWHM*_*ρ*_/*FWHM*_*z*_) as a function of detection NA for *γ* = 1. Symbols indicate theoretical and measured anisotropies of the PSFs presented in (b).

For most fluorescence microscopes excitation and detection is performed through a single lens and therefore the axial and lateral resolution increase monotonically with the NA of the objective (Fig. 2a). In this case, the identification of the maximum resolution is trivial. In LSFM in contrast the lateral and axial resolution are decoupled and can be adjusted independently by the NAs of the excitation objective (EN, axial resolution) and the detection objective (DNA, lateral resolution) (see below, calculation of the PSF in LSFM). The orthogonal configuration of an LSFM however imposes the constraint that the combined angular range of the excitation and the detection light cones can only span one hemisphere, i.e., *π* or 
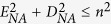
 (where *n* is the refractive index), at which point the lateral resolution can only be increased at the expense of axial resolution and *vice versa*. In practice, the available angular range is reduced by the range taken up by the lens casings. This determines the maximally available angular fill factor *γ*, according to: 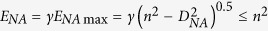 (Fig. 2c). Typical set-ups for imaging larger specimens are using - for example - excitation and detection NAs of 0.1 and 0.5 respectively, yielding an angular fill factor *γ* < 0.1. A common high-resolution set-up using two identical 0.8 NA objectives^11^ reaches a fill factor of around 0.75.

The importance of lateral versus axial resolution may vary with individual applications. Nevertheless, maximum resolution in LSFM can be universally defined as the resolution for which the focal volume defined by the effective PSF:


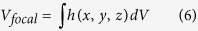


is minimal. We evaluated the integrals (Eqs 3 and 5) numerically for various pairs of orthogonally arranged detection and excitation NAs constrained by 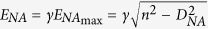; 0 ≤ *γ* ≤ 1 and calculated the effective PSF (Eq. 1). Since the lateral and axial resolution in LSFM can be strongly de-coupled, effective PSFs are compared based on the size of the resulting effective focal volume element as defined by Eq. 6. We found that the effective focal volume element reaches a minimum of about 0.023 fl for a 1.1 NA water immersion detection lens (*n* = 1.33), when complemented with a 0.75 NA excitation lens (*γ* = 1) (Fig. 2a). This is about 15% smaller than the focal volume expected for a single lens with a NA of 1.27 (maximum NA currently available for water immersion objectives). While the minimum focal volume is inversely proportional to the angular fill factor *γ* (Fig. 2d), the detection NA for which the minimum is achieved, does not dependent on *γ*. In addition, the focal volume minimum is relatively shallow thus substantial adjustments of the PSF shape, i.e., resolution anisotropy, are possible (trading lateral for axial resolution and vice versa) without substantial sacrifices on the size of the focal volume element. This means for example, that for detection NAs of 0.8–1.25 the focal volume changes by less than ~25% while the resolution anisotropy can change by a factor of ~2.5 (Fig. 2e). In fact, several systems use symmetric lens arrangements (e.g. 0.8/0.8 NA), However, the fluorescence collection efficiency and thus sensitivity increases steeply with NA, i.e., 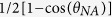. This means that a 1.1 NA lens collects ~2.2 times more light than a 0.8 NA lens. This always argues for the use of detection lenses with the highest possible NA.

Currently, the most suitable water dipping (long working distance) objective for high-resolution LSFM provides a NA of 1.1 and an access angle of ~33° (CFI75 Apo LWD 25XW, Nikon) leaving room for a maximum light-sheet illumination NA of 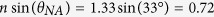. Both NAs are close to the values that provide the minimum focal volume element (see Fig. 2a). So far, the best attempt to exploit this has been by the use of a 0.65 NA custom-made objective in the standard orthogonal lens arrangement^12^, which is also used for the lattice light-sheet microscope^13^. The non-orthogonal πSPIM arrangement makes the full range accessible allowing fill factors close to unity (see section ‘*πSPIM PSF and focal volume*’ below). The use of a high NA detection lens in combination with a ultrathin Gaussian beam light-sheet is also expected to be beneficial for sensitive applications such as fluorescence fluctuation imaging (Imaging-FCS/FCCS)^11^.

### Oblique light-sheet geometry and intensity distribution

**Figure 3:**
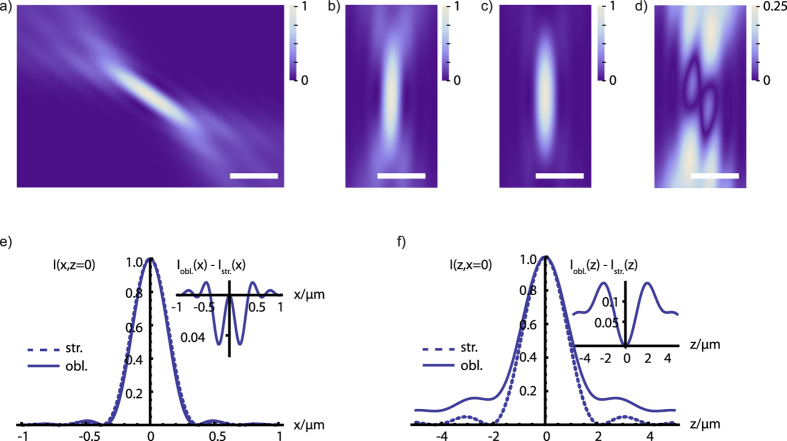
Comparison of oblique and straight light-sheet intensity distributions. Theoretical intensity distributions I(x, z) of (**a**) the oblique light-sheet (*θ*_0_ = 57.5°), (**b**) rotated version of (a). (**c**) the straight light-sheet (*θ*_0_ = 0°) intensity distribution (with *λ* = 500 *nm*, *n* = 1.52, and *θ*_*NA*_ = 32.5°). (**d**) Absolute difference between oblique and straight light-sheet intensities. Please note that the scaling of this image is different (4×, indicated by the scale bar) in order to visualize the differences between oblique and strait light-sheet better. Bars, 500 nm. Comparison of the lateral (**e**) and axial (**f**) intensity profiles for the oblique (obl.) and straight (str.) light-sheet intensity distributions in (b,c). Insets show the absolute difference between the profiles.

The non-orthogonal lens arrangement requires the generation of the light-sheet at an oblique angle so that it is aligned orthogonally to the optical detection axis (Fig. 1). The required asymmetric intensity distribution in the back-focal plane of the illumination objective (see above) leads to an asymmetric intensity distribution in the light-sheet. Using Eqs 4 and 5, we evaluated the extent of this asymmetry for our current set-up (Fig. 1i) with *θ*_0_ = 57.5° and *θ*_*NA*_ = 32.5° which is highly oblique and can thus serve as a ‘worst case scenario’. We found that the differences between oblique and straight (*θ*_0_ = 0°) light-sheet intensity distributions along the axial cross-section (see Fig. 3) are nevertheless rather small (<15% of the peak intensity). Furthermore, these differences pertain predominantly to secondary structures of the PSF and hence are expected to have no relevant effects on the imaging properties.

### πSPIM PSF and focal volume

In order to validate some of the theoretical predictions we implemented the oblique light-sheet microscope, termed *πSPIM* (for details, see ‘Methods’). We used 100 nm fluorescent beads to measured the πSPIM PSF and to determine its resolution and focal volume (see Table 1 and Fig. 2b). We found that the lateral resolution in the πSPIM light-sheet mode and the wide-field mode are comparable (284 nm and 274 nm, respectively, *λ* = 525 nm) and close to the wide-field diffraction limit (240 nm)^14^. However, the πSPIM light-sheet mode provides a substantially (~2.3 fold) higher axial resolution (339 nm) compared to the wide-field mode (785 nm), yielding a nearly isotropic PSF (FWHM_x,y_/FWHM_z_ = 0.84). This performance compares well with previous published work (see Table 2). We obtained estimates for the focal volume elements (Table 1) for the measured PSFs by a comparison of the ratios of measured and diffraction limited FWHM: 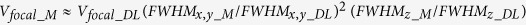, where subscripts_M and_DL are for ‘measured’ and ‘diffraction limited’, respectively. We found the focal volume for the πSPIM light-sheet mode to be approximately 1.3 fold smaller than for the wide-field mode (0.057 versus 0.072 fl) and comparable to that measured for a 1.27 NA lens, i.e., the currently highest available water immersion NA.

### πSPIM imaging of endocytosis in yeast

**Figure 4:**
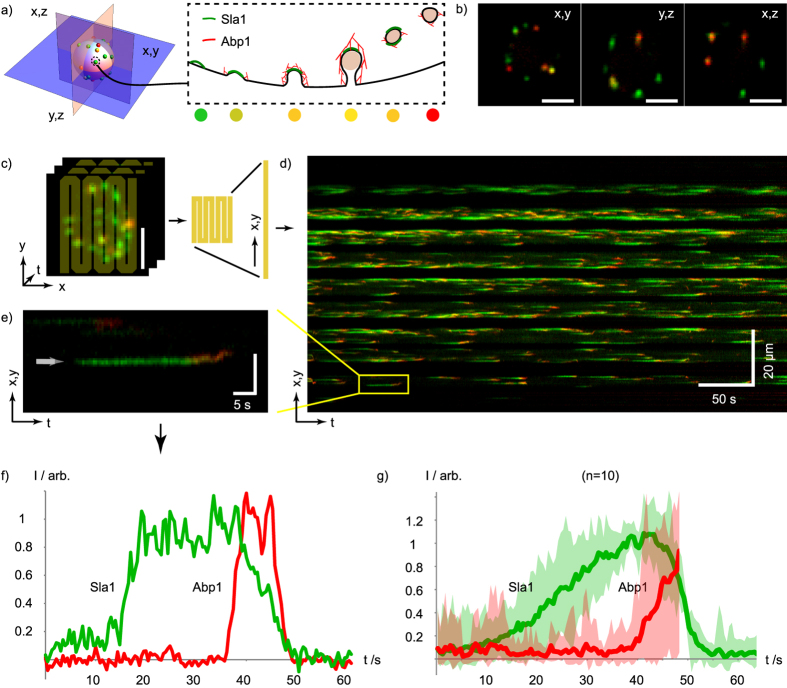
Long term *in toto* πSPIM imaging of endocytosis in yeast. (**a**) Sla1 and Abp1 are two components involved in different steps during clathrin mediated endocytic uptake of vesicles from the plasma membrane in yeast. (**b**) Mid-plane cross-section images of a yeast cell with fluorescently labeled Sla1 (Sla1-EGFP) and Abp1 (Abp1-mCherry) as indicated in (a), with 104 × 104 × 100 nm voxel sampling. (**c**) Illustration of kymograph trajectory (yellow overlay) on a time-series (435 ms interval) of maximum intensity projections of z-stack images of a yeast cell. (**d**) Kymograph of the flattened trajectory indicated in (c) showing all of the endocytic events over a duration of 7 min. (**e**) close-up and (**f**) normalized intensity profiles of a single endocytic event indicated in (d). (**g**) Average of 10 normalized intensity traces registered to a common Sla1 falling slope with the shaded bands indicating min and max intensities. Bars in (b,c,e), 2 μm. The time-series is provided online (Sup. Movie S2).

Clathrin mediated endocytosis is a highly dynamic process that is characterized by a hierarchical recruitment of factors from the cytoplasm to the plasma membrane (and vice versa) associated with the different steps of vesicle formation and the subsequent internalization of the vesicle via actin driven processes^15^,^16^,^17^. To study the dynamics of this process, differentially fluorescent protein tagged endocytic machinery components (e.g. GFP and mCherry tagged) are used and two movies are recorded with sub-second time resolution, usually employing wide-field fluorescence microscopes. Single plane imaging is usually used because of speed requirements and bleaching problems, all of which limit full cell imaging. TIRF microscopy (which provides superior sensitivity due to the absence of out-of-focus fluorescence) is used to record the events close to the surface of the cover slip^15^,^16^. Here we employ the πSPIM and Sla1-GFP (as a marker for early endocytic events) and Abp1-mCherry (as a marker for late, actin dependent events) to record ‘*in toto*’ entire cells. We demonstrate (see Fig. 4 and Sup. Movies 1 and 2) that with the πSPIM it is possible to image all individual endocytic events in a given cell over a period of up to 5–8 minutes (corresponding to hundreds of endocytic events per cell) and found a rate of 1.5 ± 0.3 events/μm^2^/min, consistent with previous measurements that report extrapolated values^16^. In the Supplement we provide additional examples of πSPIM movies showing Microtubule dynamics in HEK293T cells (Sup. Movie 3) and the dynamics of filopodia in HeLa cells (Sup. Movie 4).

#### Conclusions and Discussions

We conducted a theoretical analysis of the properties of oblique light-sheet microscopes and by employing a practical implementation of such a system – termed πSPIM – we validated that it is particularly suitable for the imaging of cells at the surface of a cover slip. We demonstrate its application in combination with wide-field fluorescence imaging of cells, which is useful if rare subpopulations of cells have to be imaged. Instead of wide-field fluorescence imaging, any other epi-fluorescence imaging modality, from confocal to spinning disc can be used in combination with πSPIM as well (including methods that utilize laser scanners for region-of-interest specific interferences, such as photobleaching). Upon identification of a suitable cell, switching to light-sheet imaging then yields an axial resolution increase, while simultaneously limiting the exposure of the sample to the section that is actually imaged. For small objects, such as yeast cells, this yields approx. 5–10 fold improvements in terms of photo bleaching; this factor is predicted to increases further with the dimension of the object under investigation. This will be in partial also beneficial for specific applications such as imaging fluorescence correlation and cross-correlation spectroscopy (imaging FCS/FCCS), which require high imaging speed and photon detection efficiency as well as a low background signal^11^.

For the yeast and tissue culture cell applications we used the πSPIM in combination with an oil immersion objective; for this the imaging quality deteriorates rapidly with increasing distance from the cover slip, making this set up not suitable for thicker samples. To overcome this we also used water objectives (e.g. a 1.27 NA 60×), e.g. for imaging of *C*. *elegans* and *Drosophila* embryos and obtained high quality images (data not shown). Nevertheless, for such samples the πSPIM is of limited use since it lacks the possibility to position the sample in a specific direction, as is the case for LSFMs that follow the classical set up.

In summary, our πSPIM set-up provides a useful light-sheet microscope with properties that match the requirements for the imaging of cells close to the surface of a cover slip, from yeast cells to adherent tissue culture cells.

## Methods

### Numerical integrations

Numerical integrations (Figs 2 and 3) were performed using the software MATHEMATICA 8 (Wolfram Research Inc.) and using the function *NIntegrate*.

### Preparation of fluorescent bead slides

Test slides were prepared using 100 nm diameter multicolor beads (TetraSpeck, T-7280, Molecular Probes). In short, the original bead suspension was sonicated using a water bath sonicator, 2.5 μl of a 10-fold diluted suspension was placed on a coverslip (24 × 24 mm, #1.5, Menzel-Gläser; washed using 100% ethanol) mounted in a 50 mm round petri-dish (351006, FALCON) (Fig. 1c) and left to dry. For imaging, the dish was filled with deionized water.

### Culturing of cells - Yeast cell culture

Cells were grown to logarithmic phase (OD_600_ = ∼0.5) in sterile filtered synthetic complete low fluorescence medium^18^. To immobilize the cells on the surface of the cover slip the slides were surface activated with BioConex (UCT, Bristol, PA) (1/100 dilution in EtOH) for 30 min at room temperature. The slide was washed once with 100% ethanol and once with water followed by incubation with 150 μl of 1 mg/ml ConA (C2010, Sigma) for 30 min and two washes with water. 50–200 μl of the cells in log phase were allowed to settle for 5–10 min followed by one exchange of the medium.

### Mammalian cell culture

HEK293T cells were grown in Dulbecco’s modified Eagle’s medium (DMEM) supplemented with 10% (v/v) fetal bovine serum (FBS) at 37 °C in 5% (v/v) CO2. 24 h after transient transfections of the HEK293T cells using 25 kDa linear polyethylenimine (Polysciences, Worrington, PA, USA) with EB3-GFP and CD3δ-mCherry^19^,^20^. For the imaging of filipodia, HeLa cells with stable integration of lifeact-GFP and H2B-mCherry^21^ cultured with 500 μg/ml G418 and 0,5 μg/ml Puromycin. Before πSPIM imaging the medium was exchanged to FluoroBriteTM DMEM (ThermoFisher Scientific, A1896702).

### πSPIM set-up and calibration

The current set-up combines epi-fluorescence wide-field microscopy with light-sheet microscopy. Light paths and arrangement of objective lenses are outlined in Fig. 1. The current set-up of the instrument supports dual color mCherry/tdTomato and GFP imaging. For wide-field excitation two high-power LEDs (M490L3 and M565L3, both Thorlabs) with clean-up filters (ET490/20, Chroma and HC584/29, Semrock) and combiner dichroic (FF560-FD01, Semrock) are used. The LEDs are imaged onto the back-focal plane of the objective (ApoTIRF 60 × 1.49 NA, Nikon or PlanApo 100 × 1.27 NA) with a magnification of around 6 using an aspherical condenser and a plan-convex lens (ACL2520U-A and LA1986-A, both Thorlabs). A variable iris diaphragm (SM1D12, Thorlabs) positioned in the back focal plane of the plan-convex lens provides an adjustable field-aperture. Excitation light for the light-sheet mode is provided by two laser (Mambo 594 nm 25 mW and MLD 488 nm 60 mW, both Cobolt) coupled into the microscope via a combiner dichroic (LM01-503-25, Semrock), an acoustic optical tuneable filter (AOTFnC-400.650-TN with controller MDSnC, AA Optoelectronic) for intensity control, and a monomode optical fiber with collimator (KineFLEX-P-1-S-405.640-0.7-1.5-P2, QIOPTIQ). The beam is expanded to 4 mm (1/e intensity width) using a telescope consisting of two achromatic lenses (AC254-075-A-ML f = 75 mm, Thorlabs and MXA20696 f = 200 mm, Nikon) and focused by an achromatic cylindrical lens (ACY254-150-A, f = 150 mm, Thorlabs) into the back focal plane of the objective lens. The beams lateral position in this plane, i.e., the angle of the light-sheet in the sample plane is adjusted by a gimbal mounted mirror positioned in the common focal plane of the telescope. The excitation mode can be switched between wide-field and light-sheet via an electronically controlled flip mirror (MFF101, Thorlabs). Excitation and emission are separated by a 2 mm thick dual-band dichroic (ZT488/594rpc, Chroma). Emission light is filtered (HC524/628, Semrock) and focused onto a CMOS camera (GS3-U3-23S6M-C, Point Grey Research Inc.) using a tube lens (MXA20696, f = 200 mm, Nikon). Samples are positioned using a custom x-y-z stage driven by three linear piezo-walk stages (SLC-1730-S, SmarAct).

In light-sheet mode, emission is detected through a second (water dipping) objective (PlApoW 25 × 1.1 NA, Nikon) orthogonal to the light-sheet whose axial position, i.e., its focal plane, is adjusted to match the light-sheet plane using another linear piezo-walk stage (SLC-2445-S, SmarAct). The objective provides a theoretical access angle of 33° but practically could only be tilted by 32.5°, allowing the use of a maximum illumination NA of about 0.71 = (1.33*sin*(32.5°)). This combination of NAs covers almost the full semicircle, i.e., ~0.97π = 2(α_NAexc._ + α_NAdet._) and minimizes the focal volume providing a nearly isotropic resolution of ~300 nm (see Results and Discussion). Emission light is split with a dichroic (H 560 LPXR, AHF), filtered (ET525/50 and ET632/60, Chroma) and focused onto separate halves of a CMOS camera (ORCA-Flash4.0 V2, Hamamatsu) using separate achromatic tube lenses with a focal length of 500 mm (G063-239-000, QIOPTIQ). The total magnification of the system is 62.5 (corresponding to 104 × 104 nm pixel). Note: separate axially adjustable tube lenses are used in order to match the focal planes of the emission channels, i.e., correct residual axial chromatic shifts of the objective lens. The two emission channels are combined in front of the camera with a second dichroic (H 560 LPXR, AHF). Fast z-stacks are acquired using a piezo-scanner (P721 PIFOC with controller E-662, Physik Instrumente). Control voltages for the various parts of the microscope are generated with an I/O card (NI PCIe-6323, National Instruments) and controlled with custom software written in LabVIEW 2012 64 bit (National Instruments).

### Image acquisition and post-processing

Bead images were acquired over a depth range of 5 μm centered on the plane of best focus with a spacing of 100 nm and analyzed using PSFj^14^. Yeast images were typically acquired over a range of 5 μm centered around the mid-plane of the cell with a spacing of 100 or 500 nm. Raw images containing both channels are split along the centerline of the CMOS chip and saved as dual-channel multipage tiff files. Time lapse image stacks were bleach corrected using Fijis build-in bleach correction function based on histogram matching^22^. The occasional hot pixel generated at the low signal end of the time lapse by this method was removed using the build-in outlier removal function.

## Additional Information

**How to cite this article**: Theer, P. *et al*. πSPIM: high NA high resolution isotropic light-sheet imaging in cell culture dishes. *Sci. Rep.* **6**, 32880; doi: 10.1038/srep32880 (2016).

## Supplementary Material

Supplementary Information

Supplementary Movie 1

Supplementary Movie 2

Supplementary Movie 3

Supplementary Movie 4

## Figures and Tables

**Table 1:** πSPIM resolution, anisotropy, and focal volumes.

Mode	Objective(s)	NA	Resolution (FMWH)/nm		Anisotropy	Focal volume/fl
*type*			*x*,*y* (*theory*)	*z* (*theory*)	(*theory*)	(*theory*)
widefield	PlanApoW25×	1.1	274 (240)	785 (801)	0.35 (0.30)	0.072 (0.057)
πSPIM	PlanApoW25× & ApoTIRF60×	1.1/1.49*	284 (212)	339 (285)	0.84 (0.74)	0.057 (0.023)
widefield	PlanApoW60×	1.27	250 (203)	648 (510)	0.39 (0.40)	0.052 (0.027)

Abbreviations: NA, numerical aperture; FWHM, full-width-at-half-maximum, anisotropy = FWHM_x,y_/FWHM_z_.

^*^The actual NA for light-sheet generation is limited by the access angle of the PlanApoW25× to ~0.71.

**Table 2:** Overview of previously published resolution performances.

Technique	Resolution (FMWH)/nm		Reference
	*x*, *y*	*z*	
4π microscopy	280	190	Bahlman *et al*.^23^
LSM	435^1^	482^1^	Capoulade *et al*.^11^
LSM (Bessel 2PE)	—	490	Planchon *et al*.^24^
Bessel plane SR-SIM	185/238	348	Gao *et al*.^12^
Lattice light-sheet	230^2^	370^2^	Chen *et al*.^13^
πSPIM	284	339	this work
widefield 1.27 NA	250	648	this work
widefield 1.49 NA (TIRF)	222	504	Theer *et al*.^14^

Abbreviations: LSM, light-sheet microscopy; 2PE, two-photon excitation; SIM, structured illumination microscopy; ^1^calculated from given 1/e^2^ radii; ^2^theoretical value.
