# Supplementary material for: πSPIM: high NA high resolution isotropic light-sheet imaging in cell culture dishes
Source: Sci Rep. 2016 Sep 13;6:32880. doi: 10.1038/srep32880 (PMC5020645; doi:10.1038/srep32880)
Supplement: Supplementary Information [file srep32880-s1.pdf]

Supplementary Information for

πSPIM: high NA high resolution isotropic light sheet imaging in cell culture dishes

Patrick Theer[1,2], Denitsa Dragneva[1] & Michael Knop[1,2,*]

[1] Zentrum für Molekulare Biologie der Universität Heidelberg (ZMBH), University of Heidelberg, Im Neuenheimer Feld 282, 69120 Heidelberg, Germany

[2] Deutsches Krebsforschungszentrum (DKFZ), Im Neuenheimer Feld 280, 69120 Heidelberg, Germany

* corresponding author: m.knop@zmbh.uni-heidelberg.de

Supplementary Movies

Supplementary Movie 1 | *In toto* imaging of endocytosis in yeast. πSPIM dual-channel time-lapse movie of endocytosis in yeast capturing all endocytic events for about 1 min at a speed of ~ 2 stacks/s (567 ms interval and 104x104x100 nm voxels) and a roughly isotropic resolution of about 300 nm. Sla1-EGFP and Abp1-mCherry were used (see Results and Discussion).

Supplementary Movie 2 | Long term *in toto* imaging of endocytosis in yeast. πSPIM dual-channel time-lapse movie of endocytosis in yeast capturing all endocytic events for about 7 min at a speed of ~ 2 stacks/s (435 ms interval and 104x104x500 nm voxels). Sla1-EGFP and Abp1-mCherry were used (see Ref[15] in main manuscript).

Supplementary Movie 3 | Microtubule dynamics in HEK293T cells. πSPIM dual-channel time-lapse movie (700 ms interval) of HEK293T cells. πSPIM dual-channel time-lapse movie (100 stacks at 700 ms interval) of HEK293T cells. The maximum intensity projection of 35 planes (500nm spacing) trough a HEK293T cell shows microtubules (in green) by visualizing the plus-end-binding protein EB3 (EB3-GFP) relative to the ER marker (CD3δ-mCherry).

Supplementary Movie 4 | Dynamics of filopodia in HeLa cells. πSPIM dual-channel 30 minute time-lapse movie (400 stacks at 4.5 s interval) of HeLa cells with stable integration of lifeact-GFP and H2B-mCherry. Maximum intensity projection of 25 planes trough a HeLa cell expressing lifeact-GFP shows the dynamics of its filopodia.
